# Numerical analysis for the vertical bearing capacity of composite pile foundation system in liquefiable soil under sine wave vibration

**DOI:** 10.1371/journal.pone.0248502

**Published:** 2021-03-17

**Authors:** Huang Zhan-fang, Xiao-hong Bai, Chao Yin, Yong-qiang Liu

**Affiliations:** 1 School of Construction Engineering, Shandong University of Technology, Zibo, Shandong, China; 2 School of Architecture and Civil Engineering, Taiyuan University of Technology, Taiyuan, Shanxi, China; China University of Mining and Technology, CHINA

## Abstract

Composite pile foundation has been widely used in ground engineering. This composite pile foundation system has complex pile-soil interactions under seismic loading. The calculation of vertical bearing capacity of composite pile foundation is still an unsolved problem if the soil around piles is partially or completely liquefied under seismic loading. We have completed indoor shaking table model tests to measure the vertical bearing capacity in a liquefiable soil foundation under seismic loading. This paper will use a numerical approach to analyze the change of this vertical bearing capacity under seismic loading. Firstly, the Goodman contact element is improved to include the Rayleigh damping. Such an improvement can well describe the reflection and absorption of seismic waves at the interface of soil and piles. Secondly, the Biot’s dynamic consolidation theory incorporated an elastoplastic model is applied to simulate the soil deformation and the generation and accumulation of pore water pressure under seismic loading. Thirdly, after verification with our indoor shaking table test data, this approach is used to investigate the effects of pile spacing on liquefaction resistance of the composite pile foundation in liquefiable soil. The time histories of pore water pressure ratio (*PPR*′) are calculated for the liquefiable soil and the vertical bearing capacity in partially liquefied soil is calculated and compared with our indoor shaking table test data at the 3D, 3.5D, 4D, 5D and 6D cases (D is the pile diameter). It is found that the pile spacing has some influence on the extent of soil liquefaction between piles. The vertical bearing capacity varies with liquefaction extent of inter-pile soil. The optimization of pile spacing varies with liquefaction extent. These results may provide some reference for the design of composite pile foundation under seismic loading.

## 1. Introduction

A composite pile foundation is different from the traditional pile foundation in its bearing capacity. The total bearing capacity of a composite pile foundation comes from the pile body itself and the soil between piles under pile cap. The design for a traditional pile foundation does not consider the contribution from the soil. The bearing capacity of a pile mainly depends on the side friction from the soil around the pile. For the liquefiable soil between piles, earthquake loading rapidly generates pore water pressure and changes the soil properties and friction force between soil and piles. That is, the pore water pressure cannot dissipate in the short time of earthquake. The increase of pore pressure makes the effective stress decrease. When the effective stress completely disappears, the soil is completely liquefied. At this time, the shear strength of soil decreases to zero and the soil is in suspension state or “liquefaction”. The liquefaction degree of the soil varies with consolidation or drainage time.

Soil liquefaction is one of the most important disasters in earthquake. During an earthquake, the bearing capacity of a pile foundation gradually decreases with the liquefaction of foundation soil. The bearing capacity reduction may lead to the collapse of buildings, bringing massive economic, life, and property losses. Therefore, the dynamic responses of a liquefiable soil-pile composite system should be carefully investigated.

Pile-soil interactions have been investigated through various laboratory tests. For example, Stanton et al. [1] conducted a shaking table test on model piles in dry sand with cylindrical, flexible containers to simulate boundary conditions. They obtained the static and dynamic responses of pile foundation and compared the responses with theoretical analysis. The actual soil shear conditions in the pile-soil dynamic responses were simulated in a large-scale shaking table test with a homemade layered shear deformation box [2–4]. Huang et al. [5] conducted shaking table tests on the seismic performance and interaction of PHC pipe-piles in saturated soils under a series of sine wave, typical earthquake waves, and some in-situ based artificial ground motions. These test results can be used to calibrate FE models and provide a database on the seismic design of PHC pipe piles. Zhang et al. [6] demonstrated that the PCP composite foundation was more effective in avoiding the mechanical resonance of the upper building. The excess pore water pressure induced by an earthquake can dissipate quickly because of the high permeability of PCPs, thus foundation liquefaction is effectively inhibited. Amar et al. [7] carried out the dynamic analysis for single and group piles under vertical vibration using the cone model. They investigated the effect of different parameters on the responses of single and group piles. In their parametric study, the effects of material damping, Poisson’s ratio, and slenderness ratio of single pile on dynamic stiffness coefficient and frequency–amplitude response are investigated. Paramasivam et al. [8] performed dynamic centrifuge tests on a 9-storey model structure with different yield capacities founded on a layered liquefiable deposit with and without a silt cap. These results show the importance of characterizing soil interlayering, structure’s strength and other dynamic properties when the consequences of liquefaction are evaluated. Al-Isawi et al. [9] developed a practical approach to extend the soil-structure interaction (SSI) database and pile performance to strong excitations. This study provided an insight into a set of SSI problems and proposed a procedure for the calibration of the advanced SSI analysis. They set up a framework to simulate a shaking table test on a model pile-foundation superstructure in soft clay. These test results provide experimental data for theoretical or numerical model validations.

Different constitutive models have been used in the pile-soil interaction analysis in liquefiable soil. Nadarajah et al. [10] used a fully coupled finite element computer code to investigate the overall response of piles in unsaturated soil. They used an elastoplastic constitutive model to represent the stress-strain behavior of soil skeleton. Zou et al. [11] used a plasticity model for the large post-liquefaction deformation of liquefiable sand and an equivalent nonlinear incremental model for stone columns (SC). They simulated a centrifuge model test with a SC-improved 19m liquefiable sand layer. The results show that the SC installation can mitigate earthquake-induced liquefaction in the saturated sand. Chatterjee [12] conducted a seismic analysis of 2⊆2 pile group embedded in liquefiable soil and underlain by non-liquefiable soil by using FLAC3D, a finite difference based geotechnical program. The pile group is subjected to vertical and lateral loads at the pile top and the earthquake motion at the pile tip. However, the actual constitutive relation of soil under dynamic load is complex and thus a universal constitutive relation is not available. At present, the dynamic nonlinear constitutive models for saturated soils under cyclic loading can be divided into two categories: one based on viscoelastic theory and the other based on elastic theory.

Theoretical study on pile-soil interaction has been conducted. Wang and Feng [13] discussed the interrelation between the attenuation of the p-y curve parameter and the soil layer cumulative pore water pressure ratio with the help of a shaking table test. Qi [14] proposed an experimental method to study the interaction between saturated sand and a model pile based on the principle of effective stress. Their experimental method can measure the residual pore water pressure of soil after vibration. Jiang [15] established the governing equations of longitudinal and horizontal vibrations of single pile in liquefied soil layer based on the dynamics of the pile-soil interaction with the Winkler foundation beam model and the Novak’s thin-layer theory. They further obtained the vibration impedance solution. Li [16] compared the fundamental dynamic response of surface motion piles in a liquefied soil layer and a non-liquefied soil layer. He found that the relative displacement of pile-soil is much larger than that of the non-liquefied soil layer displacement. Xin [17] introduced a new p-y curve based on the Winkler foundation beam theory in the shaking table test. He built a numerical model to analyze the dynamic interaction of liquefied site pile-soil bridge structure, and considered the stiffness and excess pore pressure ratio of soil around the pile. Su et al. [18] explored the dynamic behavior of a soil-pile-quay wall (SPQW) system subjected to liquefaction-induced lateral spreading in terms of experimental investigation, numerical simulation, and global sensitivity analysis (GSA). Mao [19] systematically analyzed the horizontal nonlinear dynamic response of single pile under horizontal earthquake through establishing a three-dimensional finite element geometric model of a single pile-soil system. Liu [20] studied the elastic-plastic dynamic characteristics of pile-soil-structure systems using a dynamic finite element time-history analysis. Li et al. [21] used the parameter identification method to analyze the test records of soil acceleration and pore water pressure from the shaking table tests for dynamic liquefiable soil-pile-structure interaction system. Karatzia et al. [22] studied the influence of liquefaction on the dynamic impedance (stiffness and damping) of rigid square footings resting on liquefiable soil under external harmonic loading. Their results demonstrate that liquefaction in the foundation soil yields the significant degradation of the dynamic spring coefficients and increases the associated damping coefficients under seismic excitation conditions. However, these above-mentioned investigations all focused on the horizontal bearing capacity of pile liquefied soil under horizontal vibration loading. The vertical bearing capacity of composite pile foundation system has not been investigated in liquefiable soils, especially in the case of partially liquefied soil.

Based on our previous indoor shaking table model tests [23, 24], this paper will analyze the change of this vertical bearing capacity under seismic loading through numerical simulations. Main jobs include the followings: First, the Goodman element is improved to include Rayleigh damping. This improved element can make the shear strain of the soil around the pile present a nonlinear change, and the contact element also show a nonlinear change. Second, the soil deformation and the generation and accumulation of pore water pressure under seismic loading are simulated through the Biot’s dynamic consolidation theory [25] and an elastoplastic model. Third, after the numerical model is verified with the indoor shaking table data, the time histories of pore water pressure ratio are calculated for the liquefiable soil at the 3D, 3.5D, 4D, 5D and 6D cases. The effects of pile spacing on liquefaction resistance of the composite pile foundation are further analyzed in liquefiable soil. Last, the vertical bearing capacity of pile foundations is further investigated at 0s, 15s, 25s, 35s in the process of vibration with different pile spacing.

## 2. Establishment of numerical model and computational procedure

### 2.1 Geometric model and parameters

**Fig 1:**
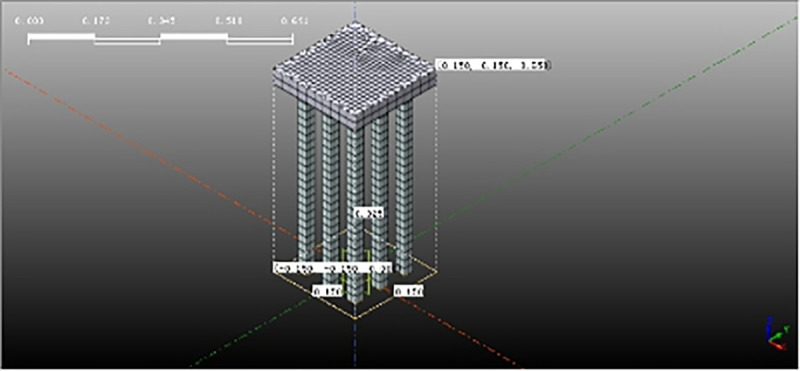
Pile and pile cap model.

The size selection of the pile and the bearing plate is basically the same as our indoor shaking table model test. Restricted by the shaking table size and load, a 10mm-thick polyethylene plate layer is pasted on the bilateral box walls towards the vibration to simulate the boundary conditions of free sites. Although the error induced by boundary conditions can be reduced, some errors still exist compared with the free site. In order to reduce the boundary conditions induced error, the numerical simulation model extended the computation boundary 2m further away from the pile-reinforced soil zone. The thickness of the bearing layer increased to 0.5m. The liquefiable soil layer is sand, and the bearing layer is silty clay. The material parameters are listed in Table 1, the relevant physical parameters are consistent with those of shaking table test and other parameters come from literature [26]. The pile size and spacing are shown in Table 2. The geometric model is shown in Fig 1.

**Table 1:** Model parameters for numerical simulations.

Physical variable	Liquefiable soil (sand)	Bearing layer soil (silty clay)	Cushion cap	Pile
Constitutive model	D-P	D-P	Elastic	Elastic
Elastic modulus (kPa)	50000	75000	200000	200000
Poisson’s ratio	0.33	0.32	0.3	0.3
Density (g/cm^3^)	1.88	1.43	2.5	2.5
Cohesion (kPa)	6	12.8	—	—
Internal friction angle (^o^)	22	16	—	—
Vertical and horizontal seepage coefficients (m/s)	1×10^-3^	1×10^-5^	—	—

**Table 2:** Pile and pile cap sizes.

Pile		Cushion cap	
Cross section (cm^2^)	2.7×2.7	Size (mm)	Distance between outer sides of side pile plus 15
Pile length (cm)	60	Thickness (mm)	30
Spacing	3D, 3.5D, 4D, 5D, 6D		

### 2.2 Basic assumptions

In computation, following assumptions are applied to saturated soils:

1. Soil is fully saturated with water. The water flow in soil obeys the Darcy’s law.
2. The pore water and soil particles are incompressible.
3. The coefficients of permeability and compressibility do not change with consolidation.
4. The vertical relative displacement between piles and soil is small.
5. No lateral deformation of soil is considered.

### 2.3 Hydro-mechanical model for soil deformation and pore water pressure generation

**Fig 2:**
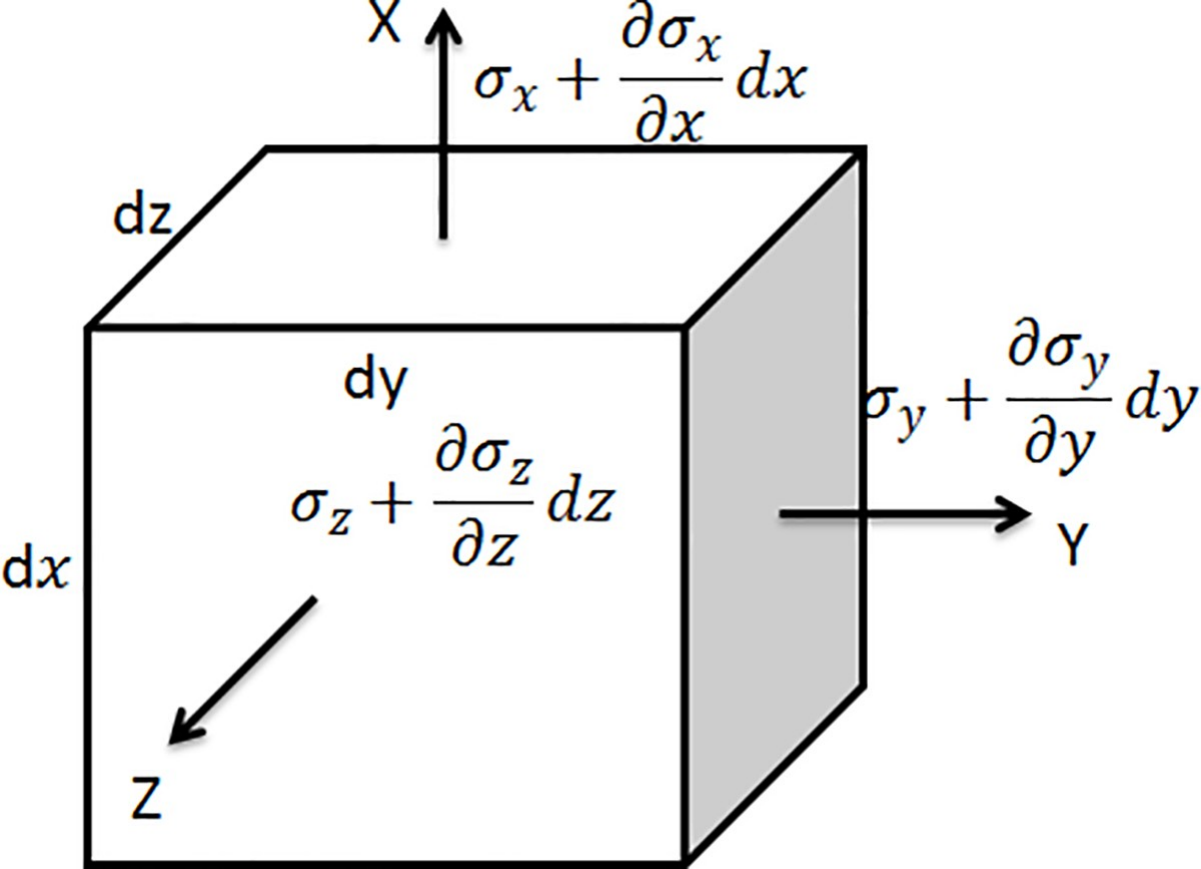
Force on a soil element.

Biot’s dynamic consolidation theory is used in this simulation. For any soil element in Fig 2, the equation of momentum is

$$\begin{array}{l} \frac{\partial \sigma_{x}}{\partial x}+\frac{\partial \tau_{xy}}{\partial y}+\frac{\partial \tau_{zy}}{\partial z}+\rho \ddot{u}=0\\ \frac{\partial \sigma_{y}}{\partial y}+\frac{\partial \tau_{zy}}{\partial z}+\frac{\partial \tau_{xy}}{\partial x}+\rho \ddot{v}=0\\ \frac{\partial \sigma_{z}}{\partial z}+\frac{\partial \tau_{xz}}{\partial x}+\frac{\partial \tau_{yz}}{\partial y}+\rho \ddot{w}-\rho g=0 \end{array}\}$$ (1)

Based on the effective stress principle, the relationship among total stress, pore pressure and effective stress is

$$\left\{\sigma \right\}=\left\{\sigma^{\prime }\right\}+\left\{p\right\}$$ (2)

The stress-strain relation can be generally expressed as

$$\left\{d\sigma^{\prime }\right\}=\left[D^{ep}\right]\left\{d\varepsilon \right\}$$ (3)

where [*D*^*ep*^] is the elastoplastic matrix of soil and *dε* is the strain increment of soil skeleton.

The interaction between water and soil is considered. The water can flow in the soil through the Darcy’s law. Under dynamic loading, the water will deform together with soil skeleton. In addition, the seepage velocity of water in soil along three directions is very small. According to Zienkiewicz’s analysis, only the gravity of water and the resistance of soil skeleton to water should be considered. Therefore, the motion of water should be

$$\begin{array}{l} \frac{\partial p}{\partial x}+\rho_{w}g\frac{v_{x}}{k_{h}}+\rho_{w}\ddot{u}=0\\ \frac{\partial p}{\partial y}+\rho_{w}g\frac{v_{y}}{k_{h}}+\rho_{w}\ddot{v}=0\\ \frac{\partial p}{\partial z}+\rho_{w}g\frac{v_{z}}{k_{v}}+\rho_{w}\ddot{w}-\rho_{w}g=0 \end{array}$$ (4)

where *k*_*v*_, *k*_*h*_ are the vertical and horizontal seepage coefficients of soil.

**Fig 3:**
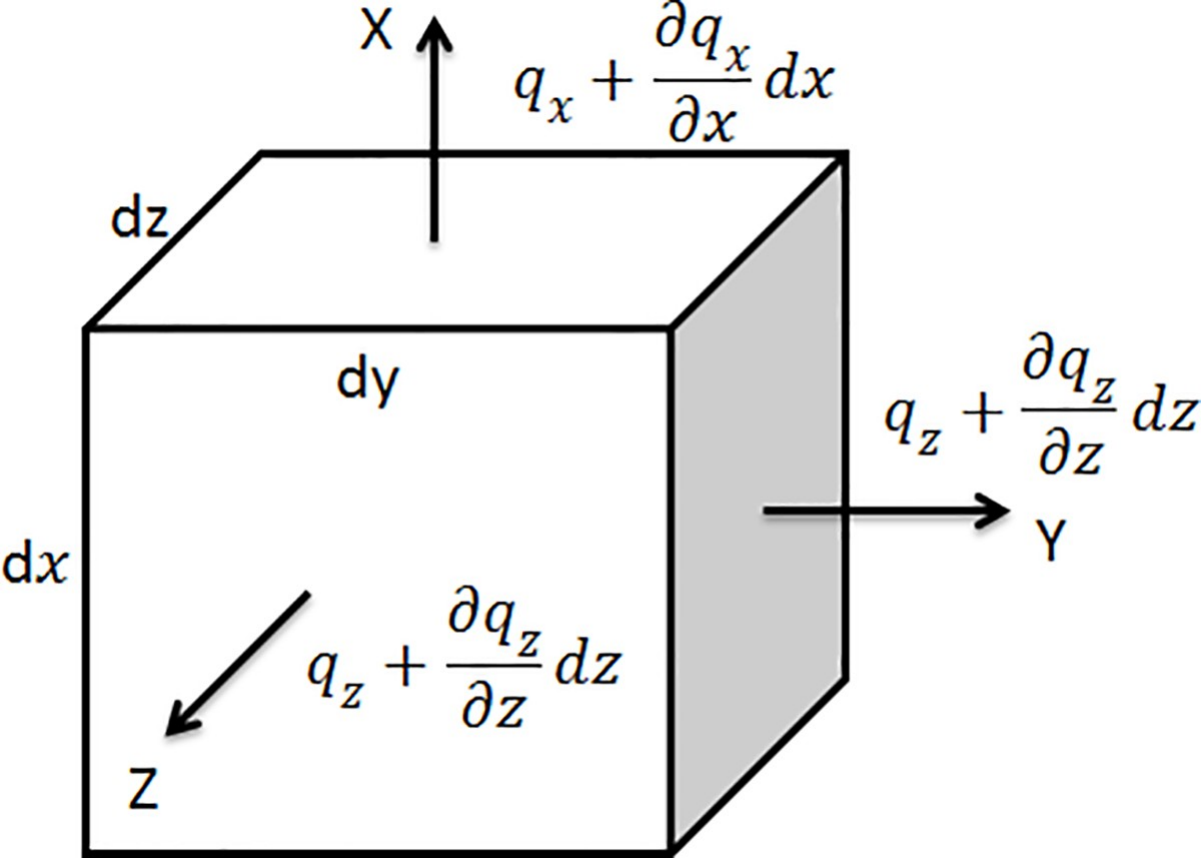
A microelement for pore water flow.

In a microelement shown in Fig 3, the water is considered to be incompressible. For saturated soil, the volume of water flowing into and out of the microelement is equal. The flow rate per unit time in three directions is q_x,_ q_y_, q_z_.

In a period of time, the soil interaction is reflected in that the flow out of the microelement may not be equal to the inflow, and the flow difference of the fluid should be the deformation of soil skeleton during the period. Thus, the condition of fluid continuity can be deduced:

$$\frac{\partial v_{x}}{\partial x}+\frac{\partial v_{y}}{\partial y}+\frac{\partial v_{z}}{\partial z}=-\frac{\partial }{\partial t}(\frac{\partial u}{\partial x}+\frac{\partial v}{\partial y}+\frac{\partial w}{\partial z})$$ (5)

The analysis and calculation for the above equilibrium equation of saturated soil are relatively stable. The pore water pressure at different vibration time can be obtained through finite element analysis, hence the liquefaction of soil can be judged based on liquefaction criterion.

### 2.4 Constitutive model for liquefiable soil

**Fig 4:**
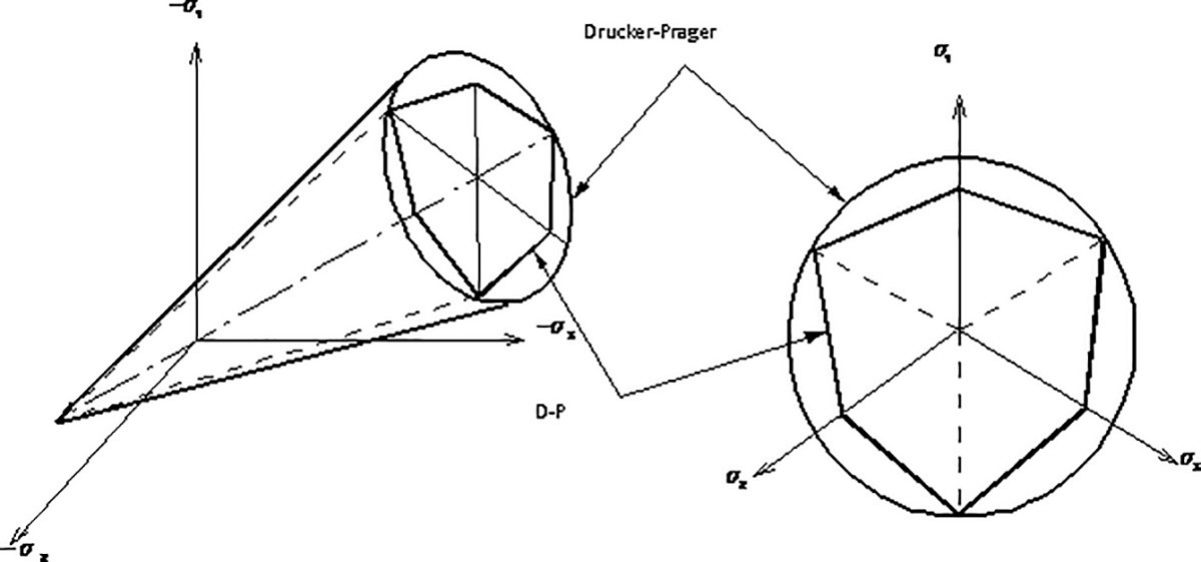
Drucker-Prager yield criterion.

The deformation of soil skeleton is described by the Drucker-Prager elastoplastic model (called D-P model later). In the principal stress space, the standard yield surface of D-P model is a conical surface as shown in Fig 4.

In the D-P model, the yield function is

$$f\left(p,\sqrt{J_{2}}\right)=3\sqrt{J_{2}}\alpha p-k=0$$ (6)

The hardening parameter is

$$H_{ij}=-3\alpha K\delta_{ij}+G\frac{1}{\sqrt{J_{2}}}s_{ij}$$ (7)

$$H=9\alpha^{2}K$$ (8)

$$J_{2}=\frac{1}{6}\left[{\left(\sigma_{x}-\sigma_{y}\right)}^{2}+{\left(\sigma_{y}-\sigma_{z}\right)}^{2}+{\left(\sigma_{z}-\sigma_{x}\right)}^{2}\right]+\tau_{xy}^{2}+\tau_{yz}^{2}+\tau_{zx}^{2}$$ (9)

Therefore, the incremental stress-strain relations is

$$d\sigma_{ij}=D_{ijkl}^{ep}d\varepsilon_{kl}=[D_{ijkl}^{e}-D_{ijkl}^{p}]d\varepsilon_{kl}$$ (10)

The elastic-plastic matrix is obtained as

$$D_{ijkl}^{ep}=(K-\frac{2}{3}G)\delta_{ij}\delta_{kl}+2G\delta_{ik}\delta_{jl}-\frac{H_{ij}H_{kl}}{H+G}$$ (11)

where G is the shear modulus and K is the bulk modulus of elasticity.

### 2.5 Damping modulus

The damping effect of soil on seismic wave is a property of soil itself, which has nothing to do with the external loading mode. In numerical calculation, the damping of soil is usually realized by establishing damping matrix depending on the stress-strain plastic relationship of soil medium. In engineering numerical analysis, “modal damping” is widely used, which gives different damping characteristics to different natural frequencies. As a kind of “modal damping”, proportional damping is widely used. In this paper, Rayleigh damping (a kind of proportional damping) is used to analyze the dynamic characteristics of soil. The damping force is proportional to the stiffness matrix of the element. In dynamic calculation, the damping matrix is obtained by orthogonal changes of mass and stiffness matrix. [*M*] and [*K*] are the mass and stiffness matrix, respectively. The damping matrix is expressed as

[C]=α[M]+β[K] (12)

$$\alpha =\frac{2\omega_{i}\omega_{k}}{\omega_{i}+\omega_{k}}\zeta ,\;\beta =\frac{2}{\omega_{i}+\omega_{k}}\zeta$$ (13)

where *ζ* is the damping ratio of the system, *ω*_*i*_, *ω*_*k*_ are the vibration frequencies. *α* and *β* are two constants. *α* is the mass factor, *β* is the stiffness factor. In this study, *α* = 0.026, *β* = 0.401 [27].

### 2.6 Contact elements between soil and structures

The structures in the soil are piles and pile cap in this study. Usually, the structure has much larger stiffness than the soil. When the soil is in the plastic state, the structural components are still in its elastic stage. This paper mainly studies the vertical bearing capacity of composite pile foundation, so it is more reasonable to adopt linear elastic model for these structures. This can meet the requirements of simulation and greatly improve the convergence speed of calculation. In the numerical model, the interface between pile and soil cannot completely transfer the deformation and stress of pile to soil. It will dissipate some part of energy. This can make the finite element solution based on energy conservation principle have error. In order to simulate the interaction between the structure and the soil under dynamic loading, this study adopts the Goodman contact element [28], a very practical contact element for the linear and nonlinear calculation of interaction problems.

**Fig 5:**
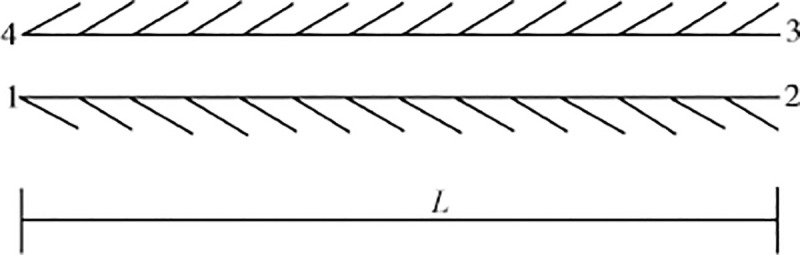
Goodman contact element.

The contact element has no thickness and mass. It connects adjacent elements through nodes (nodes 1 and 4, 2 and 3 in Fig 5) and each node has two degrees of freedom. The Goodman element was modified in this study to consider the damping or energy loss at the interface of pile-soil.

$$D=\left[\begin{array}{ccc} k_{n} & 0 & 0\\ 0 & k_{s} & 0\\ 0 & 0 & k_{s} \end{array}\right]$$ (14)

$$k_{s}=\frac{\partial \tau }{\partial \omega_{s}}=k_{1}\gamma_{w}(\frac{\sigma_{n}}{P_{a}}{)}^{n}(1-\frac{R_{f}\tau }{\sigma_{n}\tan\delta }{)}^{2}$$ (15)

*k*_*n*_ is the normal stiffness. *k*_*s*_ is the shear stiffness. *δ* is the friction coefficient between soil and structure, *γ*_*w*_ is the gravity of water, *k*_1_, n and *R*_*f*_ are nonlinear parameters of soil, *σ*_*n*_ is the normal stress, and *P*_*a*_ is the standard atmospheric pressure.

The damping term in Eq (12) will be added to the above modified Goodman element [29] in the dynamic calculation. Thus, the shear strain of the soil around the pile presents a nonlinear change. This can make the contact element perform a nonlinear change. The Goodman element between pile and soil is set as following relationship to reflect the deformation and interaction of pile and soil:

$$\left[C]=\frac{\lambda }{\omega }[D\right]$$ (16)

where *λ* is wavelength, *ω* is frequency.

**Fig 6:**
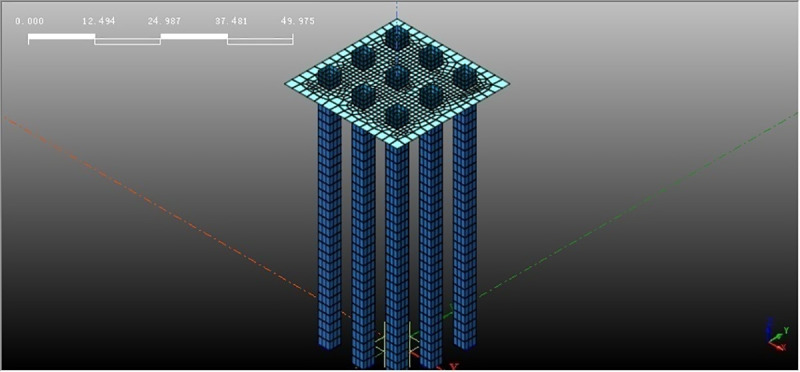
The contact elements between soil and structures.

Fig 6 presents the Goodman contact elements between soil and structures. The element parameters are listed in Table 3 [26].

**Table 3:** Parameters of various contact elements.

	Pile-soil	Cap-soil	Pile-cap
Normal stiffness K_n_ (kPa)	8000	8000	10000
Shear stiffness K_s_ (kPa)	1400	283	1500
Internal friction angle (^o^)	29	29	—
Cohesion (kPa)	78	17	—

### 2.7 Boundary conditions

The working mechanism of pile-liquefiable soil-pile cap system is analyzed under horizontal seismic wave. The boundary conditions directly affect the calculation results. When the seepage problem is involved, the seepage boundary conditions should be applied to mainly control the free degree of displacement-pore water pressure mode. For the dynamic boundary, the acceleration on the truncated boundary is assumed to be the same as the seismic wave. This method is consistent with the characteristics of shaking table test and can simulate its working conditions.

In the pile-soil interaction system under earthquake loading, the pile with higher stiffness will produce obvious external traveling wave, and the energy is transmitted outwards. If the boundary conditions are controlled by horizontal or vertical degrees of freedom, the transmission of this kind of traveling wave is prevented, and the energy can be not transmitted. In simulations, the boundary can simulate the wave transmission and the soil damping characteristics. The early viscous boundary theory is suitable for high-frequency transmission but is unstable for low-frequency. Deeks [30] proposed the viscoelastic artificial boundary for the first time. This artificial boundary can accurately simulate the radiation damping of soil on the boundary and the elastic recovery characteristics of foundation. In this method, parallel linear spring and viscous damper are added on the boundary to eliminate the reflection. This can better meet the requirements of numerical simulation.

$$Viscous\;damping\;coefficient:\;C_{b}=\rho v_{s}A$$ (17)

$$Stiffness\;coefficient\;of\;linear\;spring:\;K_{b}=\frac{G}{2r_{b}}A$$ (18)

where $\rho =\sqrt{\frac{G}{v_{s}}}$, A is the area of the element surface where the spring is applied, V_s_ is the shear wave velocity, and r_b_ is the distance from the node to the wave source.

**Fig 7:**
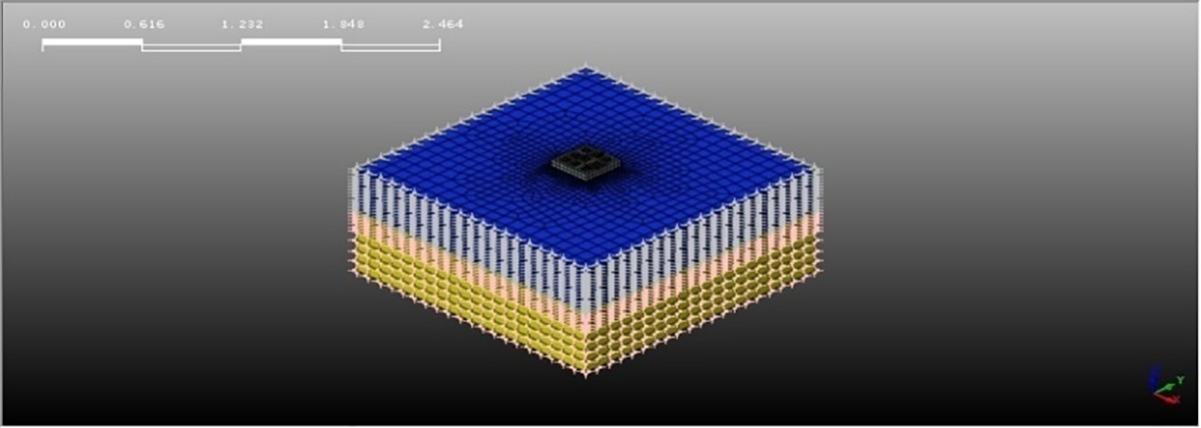
Viscoelastic boundary conditions of numerical model.

Based on the above theory, viscoelastic boundary conditions are applied to the model boundary. The shear wave velocity of the upper soil is set to 60*m/s* with the p wave velocity of 130*m/s*, with the shear wave velocity of lower soil at *280m/s* and the p wave velocity at 700*m/s*. The nodes on the model boundary are selected and the spring and damper are set on the nodes of the model boundary to generate the viscoelastic boundary, as shown in Fig 7.

### 2.8 Initial stress and initial pore water pressure

The initial stress and initial pore water pressure are important to subsequent simulations. The initial pore water pressure is applied through solving the excess pore water pressure. Firstly, the initial water pressure and the drainage boundary around the model and at the bottom are set, and the boundary conditions and the gravity field constraint to the degree of freedom are applied. The initial seepage field in the model is obtained using a seepage analysis solver. Secondly, the consolidation analysis is done with the D-P model. When the D-P model reaches the equilibrium under gravity, the consolidation initial stress field is obtained. In this way, the initial soil stress and initial pore water pressure in the foundation are obtained. Being similar to laboratory tests, the measurement and control points are set at -5cm, -30cm, and -50cm below the ground surface.

### 2.9 Sinusoidal wave input

**Fig 8:**
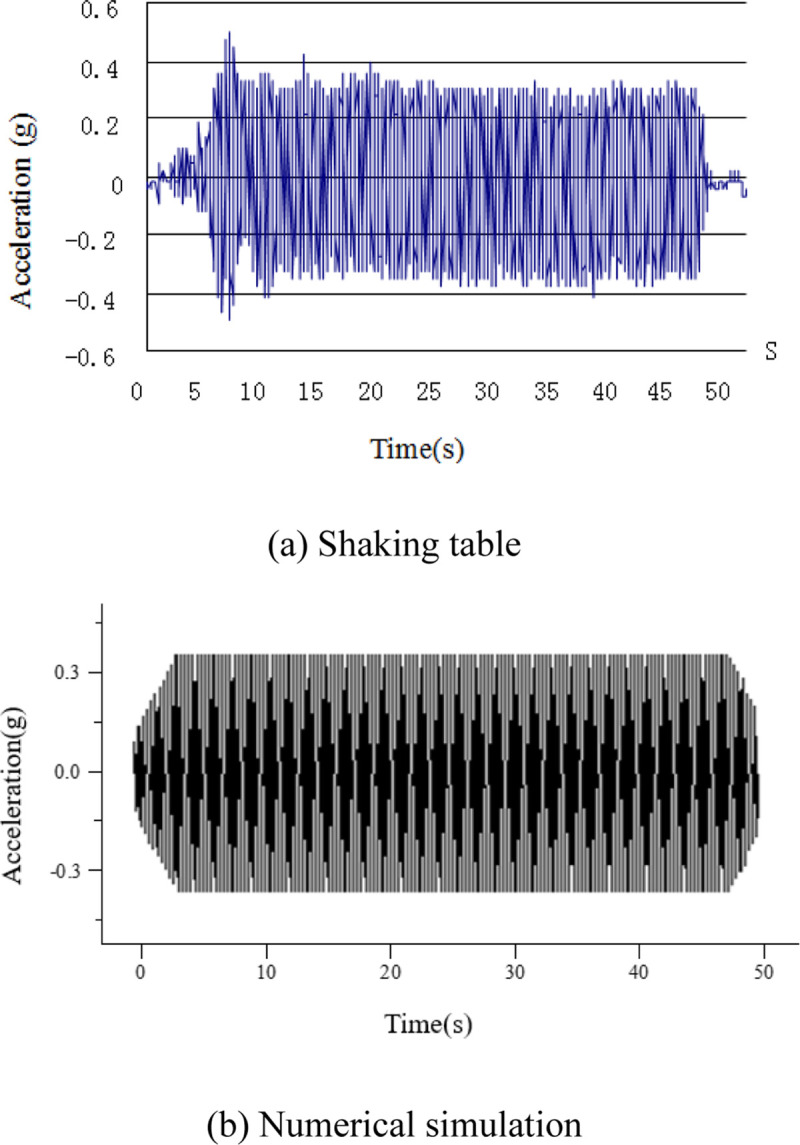
Acceleration time history. (a) Shaking table; (b) Numerical simulation.

A sinusoidal wave is input into the numerical model to simulate a seismic wave. The peak acceleration and frequency set are the same as those in our laboratory shaking table experiment, namely, *a* = 0.372g, *f* = 4.313Hz. The acceleration gradually increases to peak from 2s to 7s then lasts for 50s, see Fig 8. The acceleration time history of shaking table is obtained by acceleration sensor. At the beginning of vibration, the input acceleration is amplified by the shaking table and becomes stable.

### 2.10 Computation procedure for dynamic response analysis

The numerical computation is done with the Midas GTS [31], a finite element software for geotechnical engineering. The vertical bearing capacity of liquefiable soil composite pile foundation system is calculated through the side friction and tip resistance. For the time history analysis, more than 20 common seismic waveforms can be chosen, and the expressions of acceleration, dimensionless acceleration, displacement and velocity can be input. In the dynamic calculation, the mass unit can be converted automatically to provide an efficient operation mode for the model calculation.

Following computation procedure is used in our simulations:

1. The computational model is established in the software platform, and the initial stress and initial pore water pressure are calculated under the static condition.
2. Before the dynamic analysis, the whole dynamic time domain is divided into several smaller periods. Each period is solved individually. In the first time period, according to the dynamic constitutive relationship and initial conditions (static consolidation calculation results), the dynamic equation is solved by the step-by-step integration method. When the error between the current iteration and the previous result meets the specified requirements, the iterative calculation is finished in this period, and go to step (3); otherwise, the iteration is continued.
3. The pore water pressure of saturated sand may increase with vibration time. If the calculated average excess pore water pressure is greater than the effective stress of soil, the soil is liquefied. Before the calculation of the next period, consolidation calculation under static condition needs to be carried out again. If the liquefaction does not occur, the next calculation can be carried out directly on this basis.
4. Repeat the above steps (2) and (3) until the end of dynamic calculation.

## 3. Verification of numerical model with shaking table test

### 3.1 Initial vertical stress

**Fig 9:**
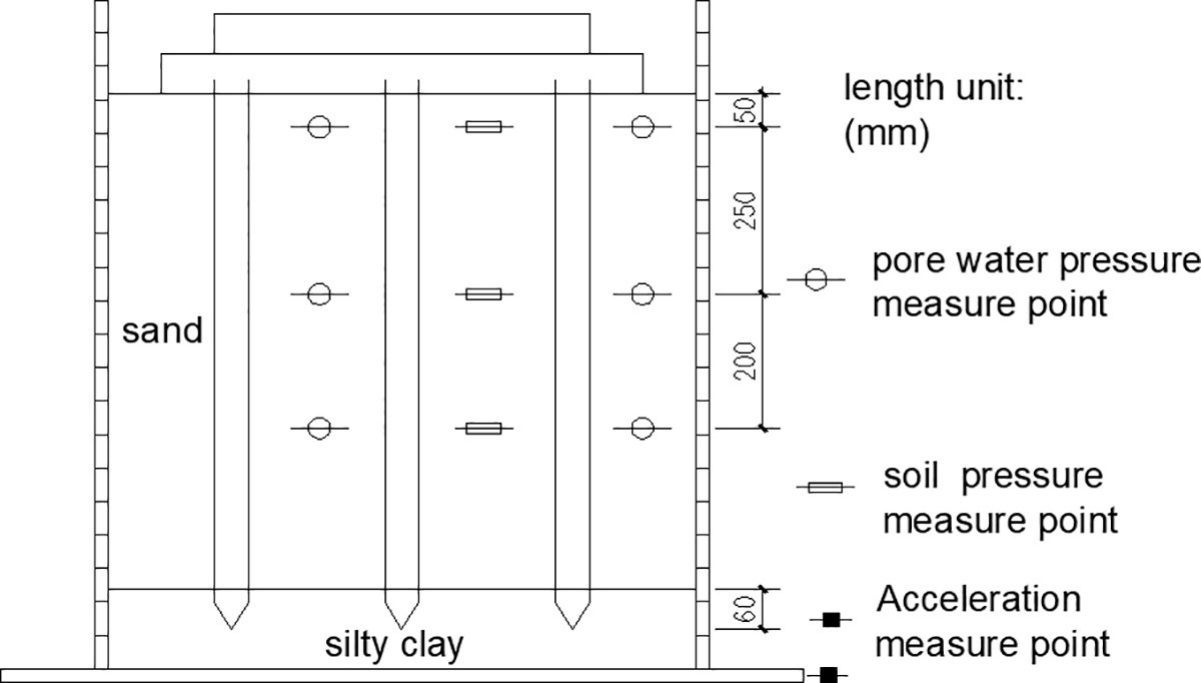
Pore water pressure and soil pressure measure points.

**Fig 10:**
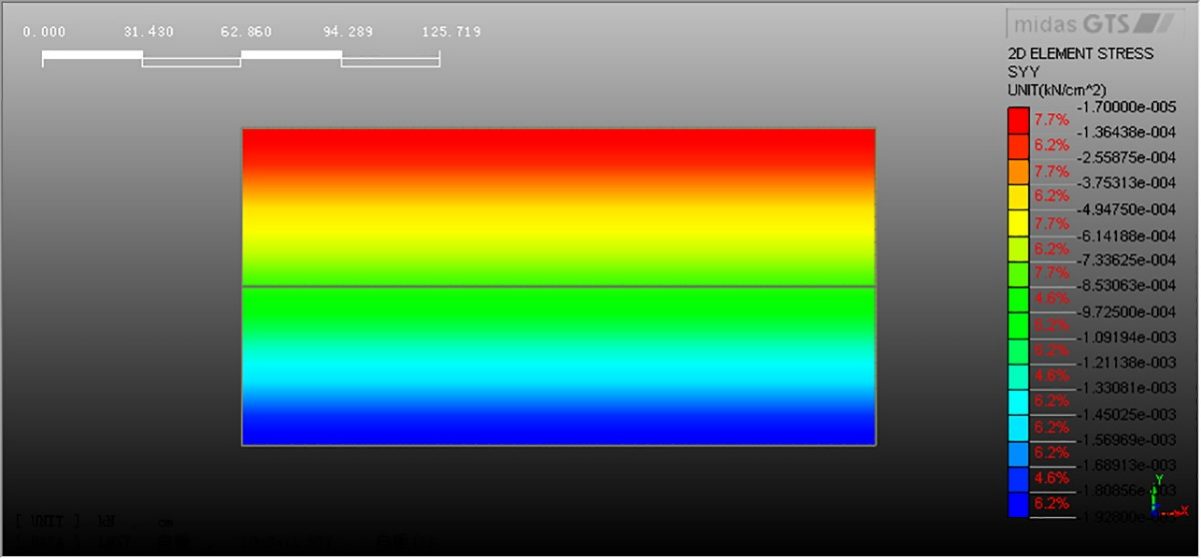
Initial stress in the natural foundation soil.

**Fig 11:**
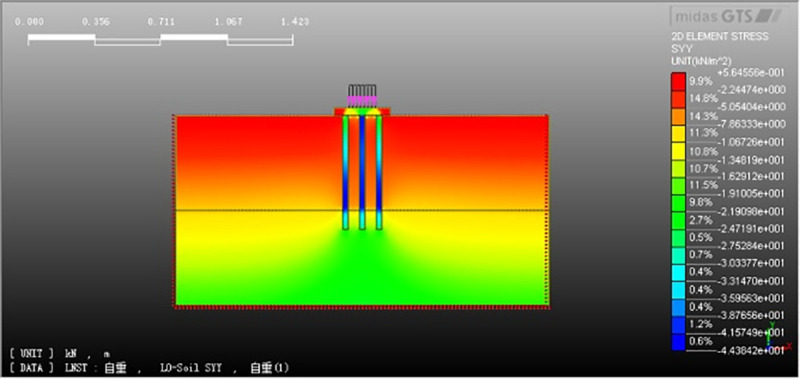
Initial stress in the composite pile foundation system.

Our previous shaking table test data are used to verify this numerical model. In the shaking table test, the initial vertical soil stress (total earth pressure) at different depths is measured by soil pressure sensor (see Fig 9). The results show that the initial soil stress at the same depth of pile foundation is different from that of natural foundation, but the initial soil stress at the same depth of pile foundation with different pile spacing is similar. In the numerical model, the corresponding initial soil stress can be obtained, see Fig 10 for natural foundation without piles and Fig 11 for composite pile foundation system. The comparison between numerical simulation and shaking table test results is shown in Table 4. Under static loading, model test and numerical simulation have relatively close initial stress. Further, the load sharing ratio of soil around the pile is similar. These indicate that the results of numerical simulation are reliable.

**Table 4:** Initial vertical stress of soil at different depths in shaking table model test and numerical simulation.

	Natural foundation			Composite pile foundation		
Depth (cm)	-5	-30	-50	-5	-30	-50
Model test (kPa)	0.457	2.739	4.565	1.098	3.260	4.903
Numerical analysis (kPa)	0.396	2.559	4.725	1.243	3.892	5.125

Note: The initial soil stress is basically the same at different pile spacing.

### 3.2 Pore water pressure ratio

Under dynamic loading, the pore water pressure in the saturated soil increases and the effective stress decreases gradually. When the pore water pressure reaches a certain value, the soil begins to liquefy. The pore water pressure ratio (PPR) can be directly used to determine the liquefaction of the soil. With the vibration time, the pore pressure ratio in the liquefiable saturated sand can better assess the soil liquefaction before analyzing the dynamic response of piles-soil. The pore pressure ratio is the specific value between the super-static pore water pressure and the initial effective stress of the soil. The excess pore water pressure *u* is the current pore water pressure minus the initial pore water pressure. A pore water pressure ratio is originally defined as

$$PPR=u/\sigma_{z0}$$ (19)

In this study, the current effective stress can be easily calculated. Thus, we propose following parameter *PPR*′ to present our results:

$${PPR}^{\prime }=u/\sigma_{zz}^{\prime }$$ (20)

where *σ*_*z*0_ is the initial stress of the soil in the vertical direction. $\sigma_{zz}^{\prime }$ is the effective stress at the current vibration time. *u* and $\sigma_{zz}^{\prime }$ are measured at the same time from the numerical simulation.

Because

$$\sigma_{z0}=\sigma_{zz}^{\prime }+u$$ (21)

Therefore,

$$\frac{1}{PPR}=1+\frac{1}{{PPR}^{\prime }}$$ (22)

**Fig 12:**
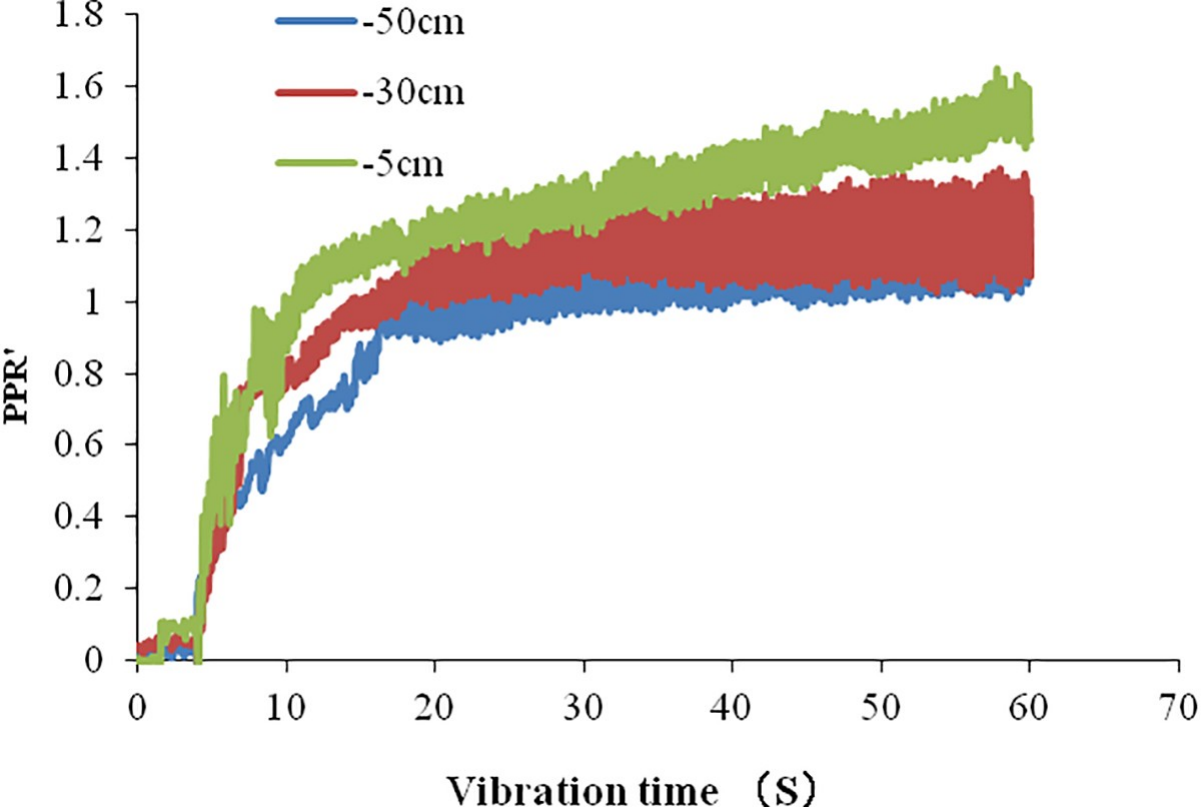
Time history of *PPR*′ at different depths of natural foundation in the shaking table test.

**Fig 13:**
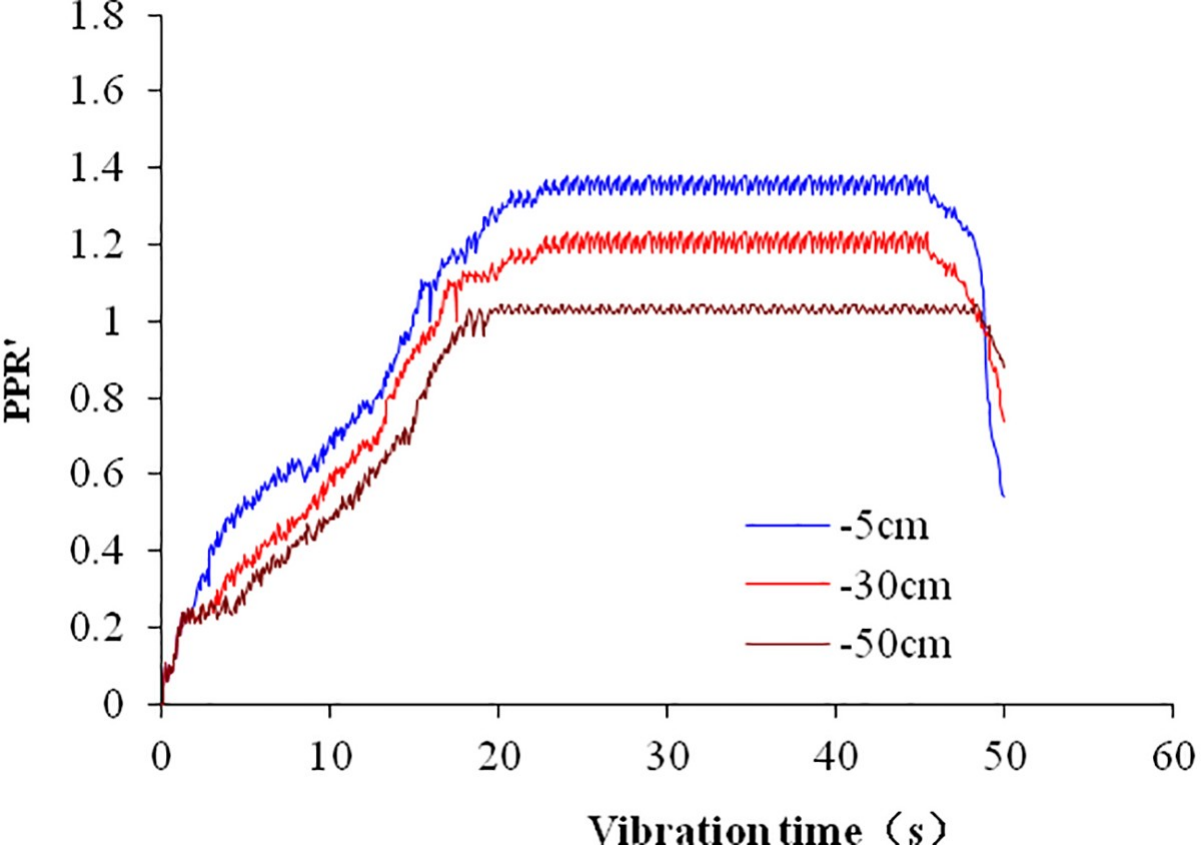
Time history of *PPR*′ of natural foundation in numerical simulation.

The natural foundation is selected to compare the time history of pore pressure ratio (*PPR*′) in the numerical simulation and shaking table test. The time history curves are shown in Fig 12 from the shaking table test and in Fig 13 from our numerical simulation. They are similar in trend. Table 5 compares the largest *PPR*′ at different depths between shaking table tests and numerical simulations.

**Table 5:** Comparison of the largest *PPR*′ in the shaking table test and numerical simulation.

	Shaking table test			Numerical simulation		
Depth (cm)	-5	-30	-50	-5	-30	-50
Largest *PPR*′	1.5	1.3	1.15	1.4	1.2	1.00

The figure shows that the value of *PPR*′ increases rapidly from 4 s to 20 s and slowly after 20 s. Both results from numerical simulation and shaking table test are similar. The largest value of PPR, at different depths is higher in the shaking table test than in numerical simulation. Their difference is about 0.1. This may be that the measured value of the pore water pressure is higher than the actual value, because the position of pore water pressure sensor moves downward in the process of vibration. On the other hand, the drainage conditions of the two methods are not exactly the same. In shaking table test, the drainage condition is not good, the measured pore water pressure is larger than numerical simulation, so the *PPR*′ is larger.

### 3.3 Soil liquefaction condition in terms of pore water pressure

In theory, reference [32] gave the condition of soil liquefaction as

σ=u (23)

where

$$\sigma =\frac{1}{3}(\sigma_{1}+{2\sigma }_{3})=\frac{1}{3}(\sigma_{1}^{\prime }+{2\sigma }_{3}^{\prime })+u$$ (24)

Thus, Eq (23) for the condition of soil liquefaction becomes

$$\frac{1}{3}(\sigma_{1}^{\prime }+2\sigma_{3}^{\prime })=0$$ (25)

For lateral constraint condition, the soil stress is

$$\sigma_{3}=k_{0}\sigma_{1},\;k_{0}=\frac{\mu }{1-\mu }$$ (26)

where *μ* is the Poisson’s ratio of soil and k_0_ is the lateral coefficient.

Further, we assume that the *σ*_1_ does not change during the whole process, therefore *σ*_1_ = *σ*_*z*0_ and Eq (23) can be expressed in terms of initial vertical effective stress as

$$\sigma =\frac{1}{3}(\frac{1+\mu }{1-\mu })\sigma_{1}=\frac{1}{3}(\frac{1+\mu }{1-\mu })\sigma_{z0}=u$$ (27)

Therefore, the pore water pressure ratio at the initiation of soil liquefaction is

$$PPR=u/\sigma_{z0}=\frac{1+\mu }{3(1-\mu )}$$ (28)

Eq (28) indicates that the *PPR* value directly depends on the Poisson’s ratio of soil. The *PPR* value in liquefaction may vary with soil and stress condition. The experimental and theoretical data from literature may verify this formula. Iwasaki et al. [33] summarized the relation between liquefaction resistance factor (FL) and PPR for 64 liquefied sites and 23 non-liquefied sites during past six earthquakes. They found that for the liquefied layers, FL decreases as PPR increases and that FL is less than 1.0 for PPR of 0.5 or higher and is more than 1.0 for PPR of 0.5 or lower. This indicates that the soil is gradually liquefied when PPR is 0.5 or higher. Furthermore, it is clarified that the sand layers are likely to completely liquefy when FL decreases to less than 0.6. Bouckovalas et al. [34] said that the compressibility of soil is essentially constant when PPR is up to about 0.5. The compressibility will be changed drastically when the PPR is 0.5 or higher. Onoue et al. [35] conducted in-situ experiments and found that the maximum PPR was measured to be less than 0.6 and suggested for PPR = 0.6 for design although their average PPR = 0.536 (Onoue [36]). Liang et al. [37] analyzed the difference of pore pressure ratio predicted by three methods and found that the maximum PPR is 0.541 for “Wu Shiming method” and 0.506 for “Onoue method”. Through shaking table tests, Wu et al. [38] found that the pile side friction drops sharply when PPR reaches 0.65. When PPR reaches 0.8, the soil completely loses the shear strength and the pile side friction almost completely disappears. The soil is completely liquefied. These results showed that soil is completely liquefied when PPR reaches 0.8. When PPR reaches 0.5 or lower, soil begins to liquefy. Hence, we can conclude that soil is partially liquefied when PPR is between 0.5 and 0.8.

As a summary, the soil in the shaking table test and numerical simulation are in partial liquefaction. The above comparison of initial vertical stress and PPR, between shaking table test and numerical simulation indicates that the numerical simulation model is applicable to the dynamic analysis for this composite pile foundation. The vertical bearing capacity of composite pile foundation during vibration can be calculated from the numerical model.

## 4. Dynamic responses of composite pile foundation under earthquake loading

### 4.1 Calculation scheme

The change of the vertical bearing capacity of composite pile foundations under dynamic loading can be difficultly observed on-site or in laboratory. However, numerical simulation can easily observe this change. The vibration time lasts for 50s. Four vibration time points of 0s, 15s, 25s, and 35s are selected to observe the change of vertical bearing capacity. When the vibration time is 0s, 15s, 25s, and 35s, the static load test is carried out to obtain the corresponding Q-S curves. The vertical bearing characteristics of piles foundation under different cases can be obtained from these Q-S curves. The calculation scheme is listed in Table 6.

**Table 6:** Controlling parameters for different working conditions.

Conditions	Data collection interval (Gravity, water head load)	Sinusoidal seismic wave	Static load
1	1s	0s	Displacement control
2	1s	15s	Displacement control
3	1s	25s	Displacement control
4	1s	35s	Displacement control

In the MIDAS GTS analysis software, the static load can be set up through the construction stage mode. Gravity is an important force in static loading. The initial pore water pressure is obtained by water table. The target load for this simulation is 5000N. The load of 750N is the load from the superstructure and is applied on the pile cap before the vibration. This is the same as the shaking table test where the pile foundation system has completed the deformation under the loading of 750N before the vibration starts. The load step is 200N from the basis of 750N. The displacement is used to control the iteration. When the displacement error is less than 0.0001mm, the iteration is terminated and the next analysis step is started. Total of 20 steps are calculated for the whole loading. When the displacement reaches 40 mm, the calculation is terminated, and the result is output.

With the change of pile spacing, the size and weight of pile cap will change. Due to the small size of the model, the weight of pile cap increases little with the change of pile spacing. This little change is not considered in this paper.

### 4.2 Effect of pile spacing on pore water pressure at different depths

**Fig 14:**
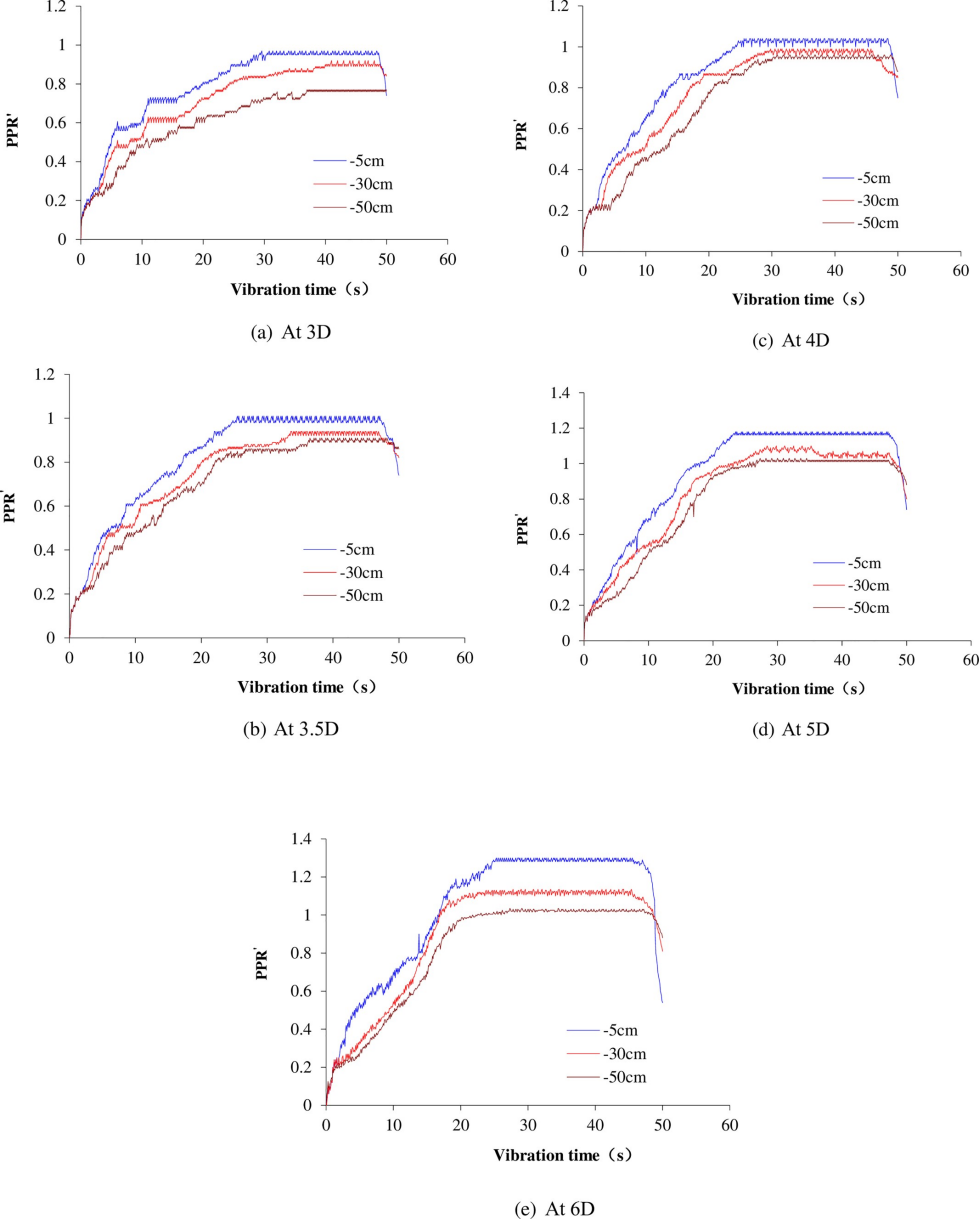
Time history of excess pore pressure ratio at different pile spacing. (a) At 3D; (b)At 3.5D; (c) At 4D; (d) At 5D; (e) At 6D.

The pile spacing is taken as 3D, 3.5D, 4D, 5D and 6D, respectively. The time history curves of *PPR*′ are presented in Fig 14. The maximum values of *PPR*′ at different depths under different pile spacing are summarized in Table 7.

**Table 7:** The maximum values of *PPR*′ at different depths and pile spacing.

Pile spacing	Soil depth		
	-5cm	-30cm	-50cm
Natural foundation	1.38	1.23	1.05
3D	0.97	0.91	0.77
3.5D	1.01	0.94	0.91
4D	1.04	0.99	0.96
5D	1.18	1.10	1.03
6D	1.38	1.14	1.03

With the accumulation of seismic wave energy, the soil liquefaction starts from the surface soil and gradually develops towards the deep soil. Actually, soil liquefaction is induced by the balance between energy accumulation and dissipation. When the two reach a dynamic balance, the pore water pressure in the soil achieves its maximum. When seismic waves stop, the energy is not accumulated anymore, and the dissipation of pore water pressure further reduces the pore pressure ratio.

Table 7 shows that the maximum *PPR*′ is 1.38 in natural foundation case. According to the Eq (22) and (28), the Poisson’s ratio is 0.27 which is basically consistent with the Poisson’s ratio of sand in Table 1. In others cases, the maximum *PPR*′ is different from the natural case due to the interaction between piles and soil. The maximum *PPR*′ is similar in 3D, 3.5D, 4D cases, but they are lower than that in the natural foundation case. The maximum *PPR*′ is higher in 5D case than in 3D, 3.5D, 4D cases. The maximum *PPR*′ of 6D case is similar to that in natural foundation case. Therefore, the piles-soil interaction has a certain influence on the *PPR*′ and the influence becomes increasingly obvious with the decrease of pile spacing.

### 4.3 Effects of pile spacing on vertical bearing capacity of composite pile foundations

In order to further investigate the dynamic interaction of piles-liquefiable soil under different pile spacing, the vertical bearing capacity of the composite pile foundation was analyzed at different vibration time.

#### 4.3.1 Variation of vertical bearing capacity with pile spacing at specific vibration time

**Fig 15:**
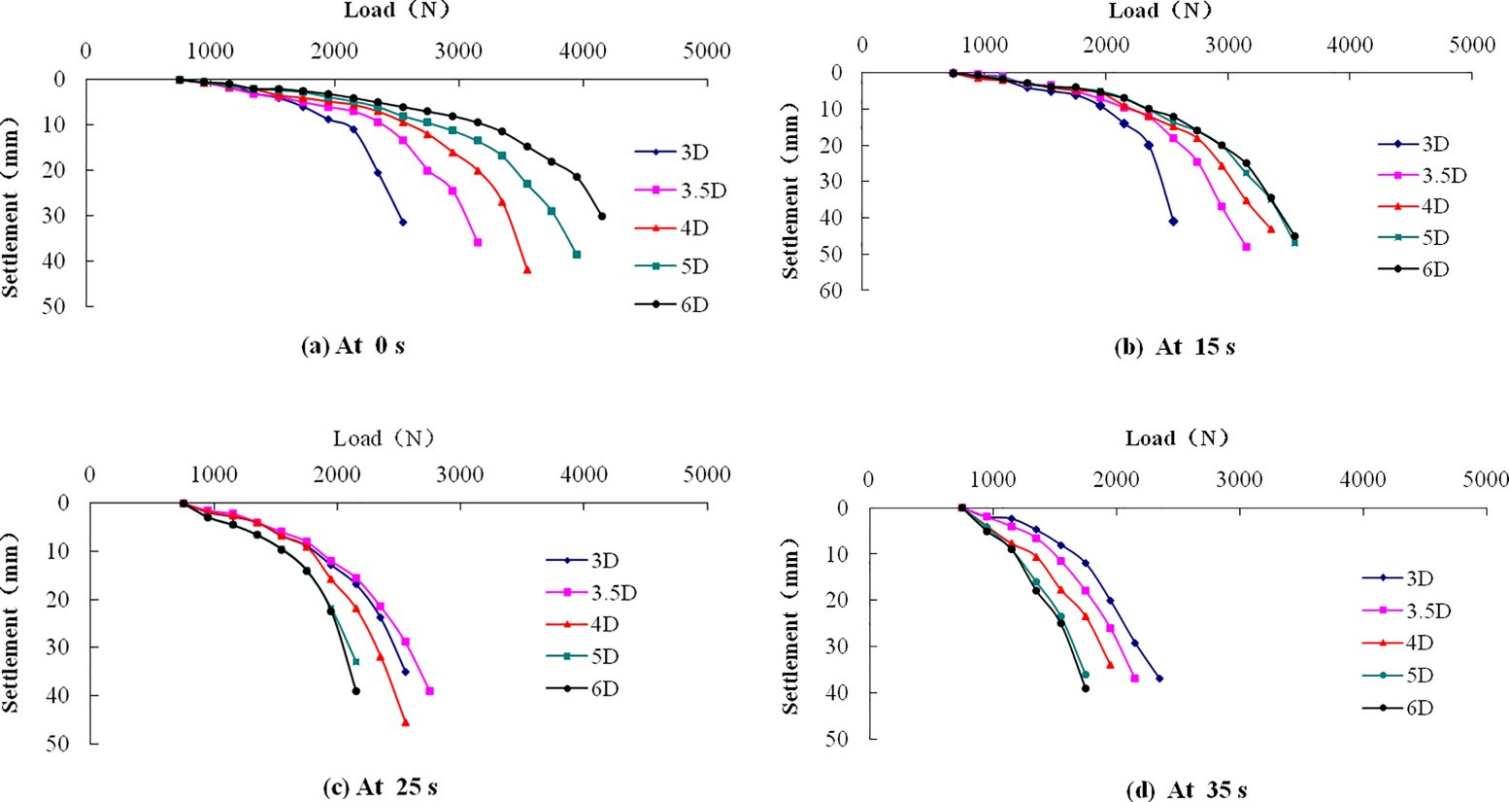
Q-S curve of the pile foundation with different pile spacing. (a) At 0 s; (b) At 15 s; (c) At 25 s; (d) At 35 s.

The static force Q-S curves of pile foundations at different pile spacing are presented in Fig 15. The vertical bearing capacity of composite piles foundation can be judged according to the following two methods [39]:

1. When the steep drop section is obvious, the load value is taken as the corresponding starting point of the steep drop section.
2. When the Q-S curve is of slow change type, the load value is taken at the total settlement of s = 40mm at pile top.

Table 8 summarizes the vertical ultimate load of the pile foundation at these specific times. The vertical ultimate load is the vertical bearing capacity of pile foundation. Before vibration time of 15s, the vertical bearing capacity gradually increases with the increase of pile spacing. This is because the pile spacing is larger, the bearing capacity of soil between piles is higher. This trend is broken with the increase of vibration time.

**Table 8:** Vertical ultimate load of composite pile foundation at specific time (Unit: N).

Conditions	0s	15s	25s	35s
3D	2200	2100	1950	1800
3.5D	2600	2450	2200	1700
4D	3100	2850	2100	1650
5D	3400	2850	1850	750
6D	3600	2800	1800	750

Particularly, followings can be observed at different vibration times:

1) Vibration time of 0s

The change trend of Q-S curve at no vibration is basically the same. The static load test shows that the vertical ultimate load of the composite pile foundation system increases gradually with the increase of pile spacing. This is mainly due to the increase of pile spacing, the range of soil between piles increases, the soil can bear more load, so the ultimate load of pile foundation is larger. When the load is less than 1800N, the curves are more compact, which shows that the vertical bearing capacity of composite piles foundation system under various cases is close to each other when the working load is small.

2) Vibration time of 15s

The Q-S curves of 0s and 15s are similar. The vertical ultimate load of composite pile foundation system increases gradually with the increase of pile spacing except the 6D case. The result in 6D case may be due to the partial liquefaction of soil between piles at the vibration time.

3) Vibration time of 25s

The Q-s curves change significantly compared with 0s and 15s. The vertical bearing capacity of 4D, 5D and 6D cases decreased rapidly. The *PPR*′ value at 5cm reaches the maximum in 4D and 5D cases at 25s. At this time, the soil is partially liquefied, but the soil around the pile can still provide a certain bearing capacity. It is noted that the *PPR*′ values at 5cm, 30cm, 50cm in 6D case all reach the maximum, and the soil between piles were liquefied in large scale. However, the *PPR*′ in 3D and 3.5D cases has not reached the maximum yet as shown in Fig 14(A) and 14(B). It is inferred that the soil has not begun to liquefy, thus the vertical bearing capacity changed little.

4) Vibration time of 35s

The Q-S curves at this time are similar to those at 25s. The curves in 5D and 6D cases have a steep drop at beginning, indicating that the composite pile foundation system has lost the vertical bearing capacity. Combining with the time history of pore water pressure ratio, it is inferred that the soil has been liquefied completely.

#### 4.3.2 Variation of vertical bearing capacity with vibration time

**Fig 16:**
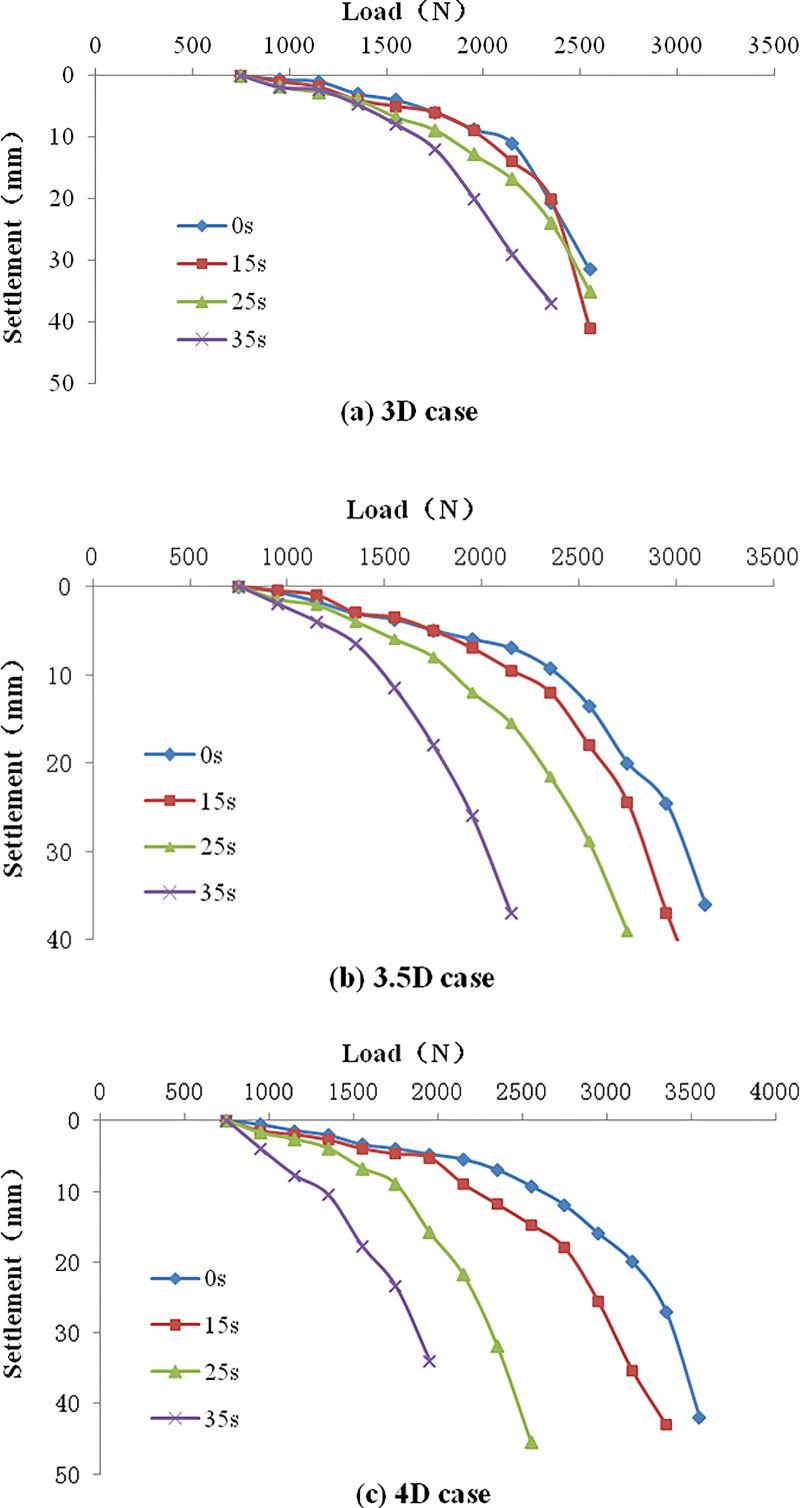
The Q-S curve of composite pile foundation. (a) 3D case; (b) 3.5D case; (c) 4D case.

Fig 16 is the load-settlement curve at different vibration times when the pile spacing is 3D, 3.5D, and 4D. In Fig 16(A), the Q-S curve gradually approaches to the vertical axis with the increase of vibration time. This indicates that the vertical bearing capacity of the pile foundation gradually decreases with the increase of vibration time. Fig 16(B) and 16(C) show the same trend. The compactness degree gradually decreases with the increase of pile spacing, thus the decreasing range of the vertical bearing capacity gradually increases with vibration time. Combining with the time history curve of pore water pressure ratio, it further shows that the anti-liquefaction resistance of the soil decreases with the increase of pile spacing.

#### 4.3.3 Change of bearing capacity with vibration time and pile spacing

Table 9 is the reduction percentage of vertical ultimate load when compared to that at 0s. This reduction percentage is the ratio of the difference between the vertical ultimate load and the 0s ultimate load to the 0s ultimate load. After 15s vibration, the reduction percentage is larger for 4D, 5D, and 6D cases than for 3D and 3.5D cases. According to the analysis of Table 6, the soil below the cushion cap for 4D, 5D, and 6D cases has been liquefied with the decreased ultimate resistance, thus pile side friction within the corresponding depth range is decreased. For 3D and 3.5D cases, the soil is not liquefied yet. The ultimate load for 3D case has not changed but 3.5D case has a slight decrease.

**Table 9:** Reduction percentage of vertical ultimate load in vibration process (%).

Conditions	0s	15s	25s	35s
3D	0	4.55	11.36	18.18
3.5D	0	5.77	15.38	34.62
4D	0	8.06	32.26	46.77
5D	0	16.17	45.59	77.94
6D	0	22.22	50.00	79.17

**Fig 17:**
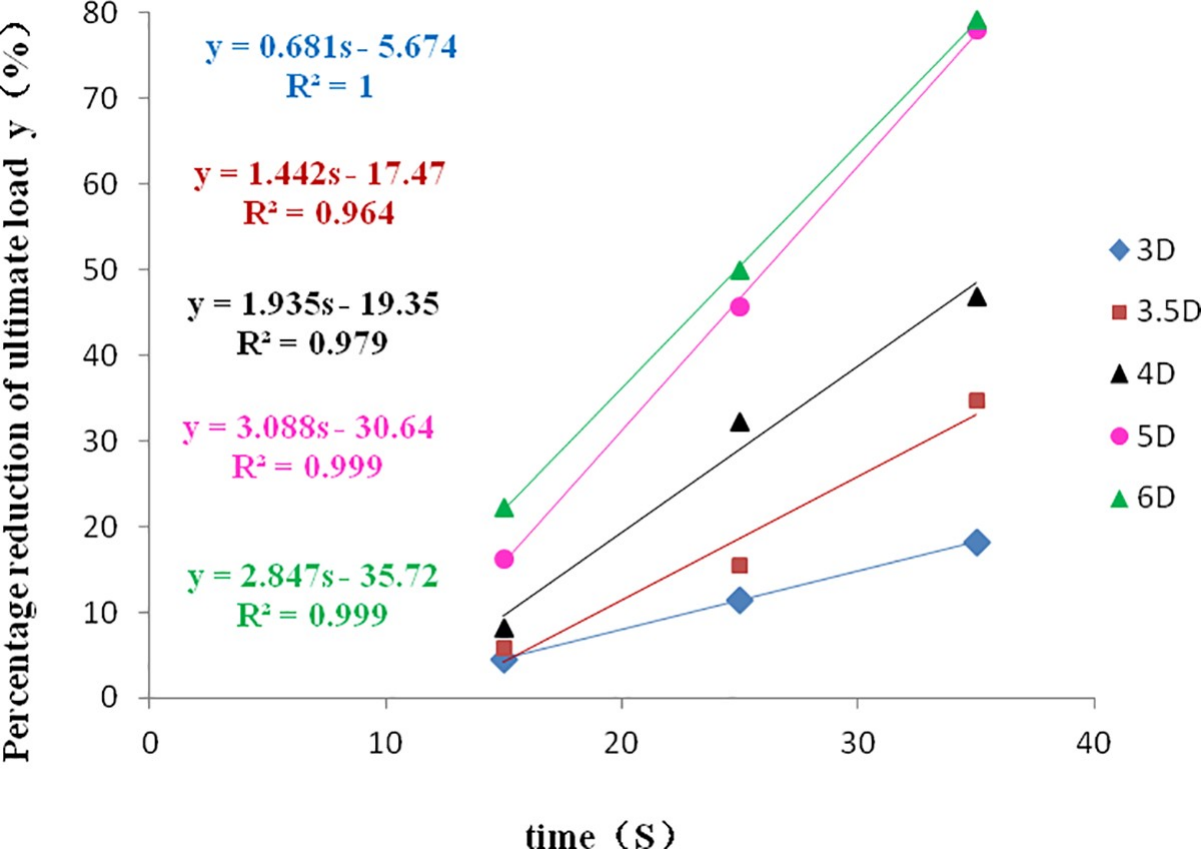
Change of ultimate load of pile foundation at different pile spacing.

Fig 17 is the fitting curve of the reduction percentage of ultimate load with vibration time at different pile spacing. For the composite pile foundation system in liquefiable sand, its vertical bearing capacity linearly decreases with vibration time and pile spacing, the decrease percentage of the ultimate load before relative vibration gradually increases at the same time of vibration. When the pile spacing is within the range of 3D-6D, the vertical bearing capacity under the dynamic load decreases gradually with the increase of pile spacing. The relationship between vertical bearing capacity and vibration time in Fig 17 can provide a reference to determine the vertical bearing capacity of pile foundation under dynamic load.

## 5. Conclusions

In this study, the vertical bearing capacity of a composite pile foundation with a low pile cap is numerically investigated in liquefiable soil system under a horizontal sine wave load. The effects of pile spacing and vibration time on the vertical bearing capacity of a composite pile foundation system are analyzed in static and dynamic loads and verified with our shaking table model test data. Based on these investigations, following conclusions can be drawn:

1. The improved Goodman contact element considering Rayleigh damping can reflect the nonlinear shear strain of soil and is able to describe the nonlinear change at the interface.
2. The soil liquefaction is a process of accumulation and dissipation of energy. The pore water pressure may increase or decrease depending on the energy balance, but the pore water pressure ratio can be used to describe the liquefaction of soil. In the whole vibration process, the pore water pressure ratio first increases gradually, then reaches a stable peak, and finally decreases gradually.
3. Pile spacing is an important parameter to affect the soil liquefaction under composite pile foundation. When the pile spacing is smaller than 6D, the composite pile foundation can improve the liquefaction resistance of the soil around piles. The 3D and 3.5D pile spacing have better resistance performance. When the pile spacing is larger than 6D, the liquefaction resistance of soil around piles is close to that of a natural foundation, thus the effect of pile foundation can be ignorable.
4. The vertical bearing capacity of the composite pile foundation is gradually weakened with vibration time. The reduction percentage of vertical bearing capacity linearly increases with vibration time and varies with pile spacing. The reduction percentage is lower for 3D and 3.5D cases than 4D, 5D and 6D cases. The weakening degree of vertical bearing capacity increases with increase of pile spacing.

Soil liquefaction is a complex phenomenon. The occurrence and development of soil liquefaction are affected by many factors such as soil properties (structure, uniformity, density, soil type, Poisson’s ratio), drainage conditions (permeability, path of seepage), dynamic conditions (acceleration, magnitude, wave form, vibration direction, frequency) and so on. This paper only investigated the influence of pile spacing on vertical bearing capacity in liquefiable soils. Other factors can be further investigated in our future study.
